# Grid codes vs. multi-scale, multi-field place codes for space

**DOI:** 10.3389/fncom.2024.1276292

**Published:** 2024-04-19

**Authors:** Robin Dietrich, Nicolai Waniek, Martin Stemmler, Alois Knoll

**Affiliations:** ^1^School of Computation, Information and Technology, Technical University of Munich, Munich, Germany; ^2^Kavli Institute for Systems Neuroscience, Norwegian University of Science and Technology, Trondheim, Norway; ^3^Department of Mathematical Sciences, Norwegian University of Science and Technology, Trondheim, Norway; ^4^Bernstein Center for Computational Neuroscience, Ludwig-Maximilians-Universität, Munich, Germany

**Keywords:** place cells, grid cells, continuous attractor networks, spatial coding, multiple scales, hippocampus, localization, evolutionary optimization

## Abstract

**Introduction:**

Recent work on bats flying over long distances has revealed that single hippocampal cells have multiple place fields of different sizes. At the network level, a multi-scale, multi-field place cell code outperforms classical single-scale, single-field place codes, yet the performance boundaries of such a code remain an open question. In particular, it is unknown how general multi-field codes compare to a highly regular grid code, in which cells form distinct modules with different scales.

**Methods:**

In this work, we address the coding properties of theoretical spatial coding models with rigorous analyses of comprehensive simulations. Starting from a multi-scale, multi-field network, we performed evolutionary optimization. The resulting multi-field networks sometimes retained the multi-scale property at the single-cell level but most often converged to a single scale, with all place fields in a given cell having the same size. We compared the results against a single-scale single-field code and a one-dimensional grid code, focusing on two main characteristics: the performance of the code itself and the dynamics of the network generating it.

**Results:**

Our simulation experiments revealed that, under normal conditions, a regular grid code outperforms all other codes with respect to decoding accuracy, achieving a given precision with fewer neurons and fields. In contrast, multi-field codes are more robust against noise and lesions, such as random drop-out of neurons, given that the significantly higher number of fields provides redundancy. Contrary to our expectations, the network dynamics of all models, from the original multi-scale models before optimization to the multi-field models that resulted from optimization, did not maintain activity bumps at their original locations when a position-specific external input was removed.

**Discussion:**

Optimized multi-field codes appear to strike a compromise between a place code and a grid code that reflects a trade-off between accurate positional encoding and robustness. Surprisingly, the recurrent neural network models we implemented and optimized for either multi- or single-scale, multi-field codes did not intrinsically produce a persistent “memory” of attractor states. These models, therefore, were not continuous attractor networks.

## 1 Introduction

Navigating large and complex environments is a non-trivial task. It requires perception of the environment, a subsequent map formed by these perceptions, a localization mechanism within it as well as a method for navigating between two points in the map (Thrun et al., 2005). Humans, as well as mammals, in general are able to perform this task seamlessly, whether in a small room or a large environment, such as a city. The neural formations responsible for the respective tasks have been investigated for decades. Yet, the exact representation a mammal keeps of an environment remains covert.

The hippocampal formation has been identified as a primary unit for the computation and storage of a neuronal spatial map ever since the discovery of PCs (PCs) by O'Keefe and Dostrovsky (1971), which was in line with the cognitive map theory by Tolman (1948). PCs were found in the CA1 and CA3 sub-regions of the Hippocampus and commonly show singular or only few prominent areas of maximal firing activity relative to the environment in which an animal is located, the cells' so-called place fields. This led to the—nowadays widely accepted—hypothesis that these neurons discretize a continuous environment into a finite number of place fields. In turn, this motivated a plethora of biological experiments as well as modeling approaches, covering a wide range of aspects, including the influence on the firing field size/shape caused by different factors, such as the environment (O'Keefe and Burgess, 1996), the animal speed (Ahmed and Mehta, 2012) or the recording location within the hippocampus (O'Keefe and Burgess, 1996). These studies revealed that place cells can express multiple place fields under certain circumstances (Kjelstrup et al., 2008; Davidson et al., 2009; Park et al., 2011; Rich et al., 2014) and that the size of these fields can vary (O'Keefe and Burgess, 1996; Fenton et al., 2008). The majority of these experiments were, however, conducted in small, confined spaces, since the technology and hardware that is required for neural recordings did not support large and unconfined environments at the time of the studies.

The advancement of hippocampal recording technology toward wireless communication recently allowed to conduct experiments in large-scale environments and to study different firing properties of PCs (PCs) in dorsal CA1 of the hippocampus in such surroundings (Eliav et al., 2021; Harland et al., 2021). Both studies reported place cells with multiple, differently sized place fields—a MSMF *(MSMF) place code*. This code is similar to the *grid code* produced by grid cells found in the (MEC; Hafting et al., 2005). While each grid cell also maintains multiple fields, the size of these fields is constant per neuron and only changes across so-called modules of neurons with the same scale (Stensola et al., 2012). The fields are distributed regularly in a hexagonal pattern forming an optimal code for arbitrary spaces (Mathis et al., 2015). In contrast to that, the experiments performed by Eliav et al. (2021) revealed the MSMF code for neurons in the hippocampus of bats flying through a one-dimensional, 200m long tunnel. Harland et al. (2021) identified the same multi-scale multi-field property for PCs in rats foraging within a two-dimensional, 18.6m^2^ open arena.

To gain further insight, Eliav et al. (2021) performed a theoretical analysis to demonstrate the effectiveness of a multi-scale code compared to other codes, including a single-scale code. In order to achieve a localization error of <2m, the authors show that a single-field model requires more than 20 times as many neurons than an MSMF model. This analysis further shows that using a fixed number of 50 neurons, the decoding error is 100 times better with the MSMF model than with the single-field model.

**Figure 1:**
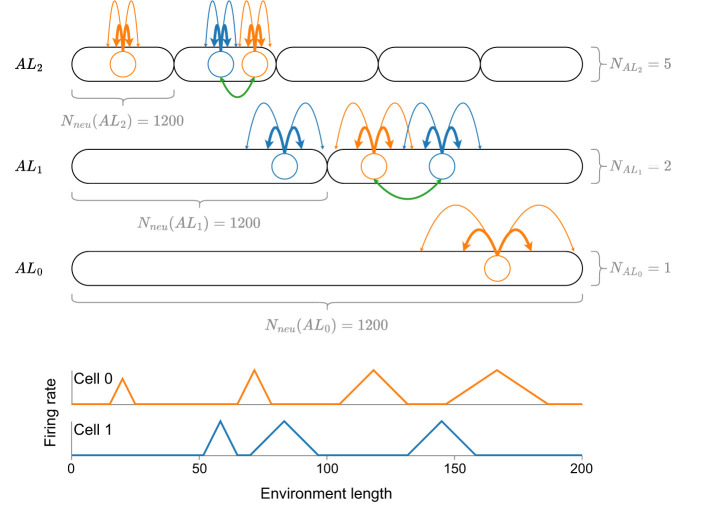
A visualization of the CAN model introduced by Eliav et al. (2021) with a total of eight attractor networks, coupled together by neurons in the same attractor (green lines). Each attractor network consists of the same amount of neurons (*N*_*neu*_ = 1, 200), drawn randomly from a total number of 4,000 neurons. At the bottom of the figure, the idealized firing rate for each of the two neurons (blue and orange) is shown. Note that, although the size of a firing field is generally pre-determined by the respective attractor network, it can vary depending on the overall connectivity of the neuron. See the first two fields of cell 0 for an example.

Beyond this theoretical analysis, Eliav et al. (2021) also introduce two neuronal models in a computational analysis, which could explain how MSMF code might be generated—a CAN (CAN) and a feedforward model receiving input from either CA3 place cells or MEC (MEC) grid cells. The 1D CAN consists of multiple, distinct, differently sized, overlapping attractor networks, each of which contains the same number of neurons, as shown in Figure 1. The authors perform simulated experiments of this network in a 200m long environment using 4,000 neurons (1,200 randomly sampled neurons per attractor) and show that this network is capable of generating an MSMF code. The analysis of this model, however, is not exhaustive. The field sizes were analyzed, as shown in Figure 1, but no experiments were reported that evaluated the decoding accuracy of said network.

Eliav et al. (2021)'s theoretical and computational analysis of the MSMF code suggests that nature has discovered a superior coding scheme for the position of an animal. Yet these results raise several important neuroscientific and computational questions. First, it has been shown previously, that the “traditional” single-scale, single-field place code is outperformed by the grid code (Mathis et al., 2012) and that such grid codes also maintain an optimal distribution of fields per neuron for arbitrary spaces (Mathis et al., 2015). These grid codes, however, are not observed in very large environments (Ginosar et al., 2021), raising the question of whether the generative mechanism for creating the grid representations fails or whether, indeed, the MSMF code has advantages over the grid code in terms of decoding accuracy, energy consumption or robustness. Second, the discrepancy between the number of neurons used for the theoretical (50) as well as the computational analysis (4,000) by Eliav et al. (2021) is non-negligible and opens up the question of whether realistic networks and interconnections would be able to achieve such a performance. Can an optimization algorithm find a network with an accuracy close to the one from the theoretical experiments? How would the neurons have to be connected? What would an optimal distribution of the fields look like? Finally, when inspecting the general structure of the original MSMF network in combination with the distribution of the fields in the experiments, one naturally wonders about the dynamics of a network for such a code. How do the coupled attractors in the MSMF network interact and interfere with each other? Would this still be a continuous or rather a discrete attractor network?

We will try to answer some of these questions in this paper using evolutionary optimization of two different MF (MF) networks. We compare the performance of several candidate networks under different scenarios and analyze them from two distinct viewpoints. First, we investigate the (dis-)advantages and properties of the codes produced by the respective networks, independently of whether the networks generating them are biologically plausible. The goal here is to identify and compare the properties of the different codes [MSMF (MSMF), SSMF (SSMF), SSSF (SSSF), and grid]. The second aspect of our study is whether the networks have continuous attractors, as has been proposed for many different brain areas (Khona and Fiete, 2022). The newly introduced multi-attractor network by Eliav et al. (2021) might be an example of a CAN network. Therefore, we evaluate and analyze the biologically relevant properties of these networks. The main contributions of our work can be summarized as follows:

- We perform an in-depth analysis of the MSMF CAN model proposed by Eliav et al. (2021), and analyze a second, more flexible CAN model that we derived from their theoretical framework;
- We apply evolutionary optimization on the parameters for both attractor network models above;
- We demonstrate that while some optimized models do work with mixed field sizes, they tend to achieve higher decoding accuracy when constructed of many small fields instead of a variety of field sizes. This is at variance with the theoretical analysis reported in Eliav et al. (2021);
- We show that a simple grid code outperforms randomly organized MF models with respect to decoding accuracy, at least in the absence of noise. The experimental observation of MF codes, therefore, might indicate that they have other advantages that go beyond the mere precision of encoding the animal's position;
- Indeed, we demonstrate that MF models are significantly more robust against noise compared to grid field as well as single field models;
- We show that lateral connections in both MF models do not form the basis of an actual CAN, but they do improve the decoding accuracy under specific circumstances,
- Finally, we provide an openly accessible framework for optimizing and evaluating the different networks^1^.

## 2 Methods

Within this section, we describe the different network models used in our simulation and optimization experiments as well as the corresponding optimization algorithms.

We start by defining different model classes used throughout this paper. There are two key determinants that we use: the number of fields that each neuron has and the sizes of these fields. In particular, neurons in the most complex model have multiple fields that come in multiple sizes. With these determinants in mind, we have the following model classes:

**Single-Scale Single-Field Model (SSSF):** Each neuron has exactly one firing field representing one location in the environment. All firing fields are of the same size.
**Single-Scale Multi-Field Model (SSMF):** Each neuron has more than one field, but the field sizes obey a unimodal distribution.
**Multi-Scale Multi-Field Model (MSMF):** Neurons have more than one field and the field size distribution has at least two separate peaks (multimodality).
**Multi-Field Model (MF):** Neurons have more than one field, but the field sizes could have any distribution (including unimodal distributions).

Throughout this paper we optimize the parameters of different MF networks. The optimized networks are then classified as either SSMF or MSMF model.

### 2.1 Network models

In the following, we describe two MF models, together with the grid field and single field model for comparison. An overview of each network's parameters is given in Supplementary Table 6. The dynamics and neuron models are identical for all networks and will be described in Section 2.2.

#### 2.1.1 Fixed multi-field model

The first MF model we consider is adapted from Eliav et al. (2021). The authors introduce a network for 1D environments, in which the neurons are organized not just in a single line attractor, but in multiple, differently-sized line attractors that interact with each other. We call this a *fixed* MF network (F-MF), due to the fixed, predetermined number of line attractors. A schematic of this architecture is visualized in Figure 1. The network consists of multiple, distinct attractor subnetworks (black ovals), distributed over three different levels. Each attractor level (*AL*_*i*_) maintains a different interaction length *L*_*int*_ for all line attractors on its level. *L*_*int*_ is the maximum distance over which two neurons maintain a positively weighted connection. In alignment with Eliav et al. (2021), we set the interaction length to be 0.05 (5%) of the size of the environment that one line attractor subnetwork covers.

As shown in Figure 1, the attractor scales are organized hierarchically, while the number of neurons per attractor stays constant (Eliav et al., 2021). Starting with a pool of *N*_*neu*_ = 4, 000 neurons, each neuron participates in each of the attractors with a probability *P*_*att*_ = 0.3. While Eliav et al. (2021) do perform some general analysis of this model (field sizes, distribution) they do not investigate the performance (positional decoding accuracy) or efficiency (potential energy consumption, number of neurons) of the network as they did in their theoretical analysis.

The default parameters used in our simulation experiments for the field and attractor generation of this model are listed in Supplementary Table 6. Most of these parameters are identical to the ones used by Eliav et al. (2021). It is unclear if the parameters reported by Eliav et al. (2021) were selected to stabilize the network, or if they were extracted from real-world recordings. For further details regarding this model, we refer the reader to Eliav et al. (2021).

One of the key questions we seek to address in this study is whether MSMF-like properties emerge naturally without *a priori* specifying subintervals of the environment to which the attractors are tuned. For this purpose, we next define a more flexible and dynamic MF model.

#### 2.1.2 Dynamic multi-field model

Based on the insights from Eliav et al. (2021), we developed a new *dynamic* MF model, (D-MF) composed of a dynamic number of attractor networks. The model has the general architecture of a CAN but does not fully comply with the properties of either a continuous or a discrete attractor network, settling somewhere in between. The core idea is that, similar to the F-MF model, each neuron can have multiple fields resulting from its participation in multiple attractors, but connections between two neurons are made dynamically, only when their field sizes are similar. This approach generalizes the concept of multiple, interacting attractors proposed by Eliav et al. (2021), for which these authors created precisely three levels of field sizes, or attractors; furthermore, these fields uniformly spanned a subinterval of the environment. In contrast, the D-MF model is capable of producing a much larger number of attractors. Depending on the parameter choices governing the connections, a MSMF as well as a SSMF model could result.

**Figure 2:**
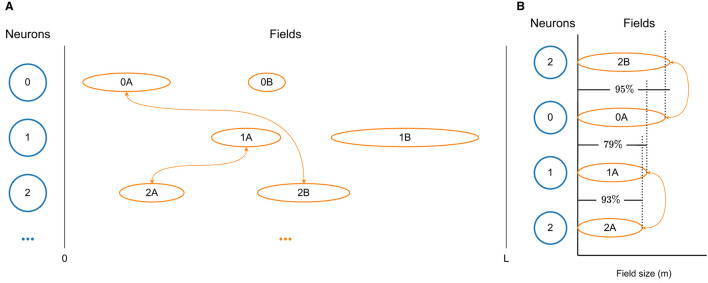
Visualizations of the D-MF model developed by us based on the theoretical model from Eliav et al. (2021). **(A)** The differently sized firing fields of three neurons. Only connections between neurons with fields of similar size (0A↔2B, 1A↔2A) are modeled. **(B)** The size difference between the firing fields, shown in detail. In this example a threshold *TH*_*fsr*_ = 0.9 = 90% was selected.

A visualization of a few neurons, together with their fields and respective connections, taken from a D-MF network, are shown in Figure 2A. In order to generate such a network, we first create a population of *N*_*neu*_ neurons and then sample fields for each of the neurons, using the same gamma distribution as Eliav et al. (2021) did for their theoretical analysis. We base the field distribution on these results, which in turn are based on their measured experimental values. New fields for a neuron continue to be generated until the overall size Σ*fs* of all fields of a neuron *n* reaches a certain threshold ${\bar{\Sigma }}_{fs}$, the value of which we took from the supplementary material of Eliav et al. (2021).

Subsequently, the connection weights between all neurons are assigned. For this purpose, we define a threshold *TH*_*fsr*_ for the ratio between the size of two fields. We then compare the sizes of all fields of two neurons (*n*_0_, *n*_1_). The overall connection strength between these two neurons is generally defined by the distance between all relevant fields of these neurons, in the same way as the connection weight for the F-MF model is calculated (see Section 2.2). In order to achieve a similar architecture as Eliav et al. (2021) with their CAN model, we only take those fields into account, whose ratio is above the threshold *TH*_*fsr*_, i.e.,


(1) $$\begin{array}{l} \frac{\text{min}\left(fs_{0},fs_{1}\right)}{\text{max}\left(fs_{0},fs_{1}\right)}>TH_{fsr} \end{array}$$

for fields with sizes *fs*_0_∈*n*_0_ and *fs*_1_∈*n*_1_. A simplified diagram of this mechanism for connection weight calculation in a D-MF network is visualized in Figure 2B. Here a threshold of *TH*_*fsr*_ = 0.9 was chosen, which means that only two connections between the three depicted neurons will be created. The first synapse connects neurons *n*_0_ and *n*_2_ with a weight based on fields *f*_0*A*_ and *f*_2*B*_. The second synapse connects neurons *n*_1_ and *n*_2_ with a weight based on fields *f*_1*A*_ and *f*_2*A*_.

In the F-MF model, this field size constraint is inherently present through the design choice of a fixed number of line attractors per level. While the F-MF model also creates multiple connections between two neurons based on the field sizes of two neurons, connections in the F-MF model are more strict in that only neurons with fields of exactly the same size (interaction length) and within the same line attractor are considered for the overall connection strength between two neurons. In the D-MF model, we only restrict the connection between two neurons based on the sizes of their respective fields using a less strict rule (see Equation 1). This connection scheme is therefore a generalization of the F-MF model and hence also uses the same weight calculation mechanism as introduced by Eliav et al. (2021) for the F-MF model.

Throughout our experiments, we use the D-MF model in order to further investigate the influence of the field size on the connection probability between two neurons and the benefits of a field size dependent connection scheme. For this purpose, we introduce a parameter that can be used to set the *field connection probability* *P*_*fc*_ directly, instead of an indirect influence by the *TH*_*fsr*_ parameter. If *P*_*fc*_ is used, the connection between two neurons is established randomly with the probability *P*_*fc*_. In this way, we can directly influence the overall fraction of connections being created, independently from the field sizes of the individual neurons. We use this parameter in our evaluation in order to identify the benefit of connecting neurons based on their field sizes or randomly.

The overall difference between the F-MF and the D-MF model is the distribution of the field sizes and the fact that in the F-MF model all attractors span (a part of) the environment uniformly. In the D-MF model, this is not necessarily the case. With the dynamic procedure for creating fields, connections, and hence attractors the position of a field within an attractor is not predetermined. The parameters used for generating the fields are listed in Supplementary Table 6. The dynamics of the network are the same as for the F-MF network and are described in Section 2.2.

#### 2.1.3 Single-scale single-field model

As a baseline, we implemented a simple single-scale single-field (SSSF) model. This model is based on the F-MF model described in Section 2.1.1, but simplified to have only one attractor level with a single line attractor spanning over the entire environment. Each neuron maintains a single firing field, while the fields are distributed uniformly within the line attractor and hence the environment.

#### 2.1.4 Grid cell model

We also implemented a one-dimensional grid model without lateral connections. This model consists of multiple modules *N*_*mod*_, each containing a fixed number of neurons $N_{neu}^{mod}$. Furthermore, each module has a certain scale, starting with the minimum defined scale $S_{mod}^{min}$ and increasing per module by the module scale factor *S*_*mod*_. The neurons within each module then maintain regularly recurring firing fields on this length scale. Across the module, different spatial offsets characterize each neuron's firing fields relative to the firing fields of other neurons in the module, thereby generating a 1D ensemble grid code. This model can be viewed as a structured version of the MF networks introduced in Sections 2.1.1. and 2.1.2. Both network types maintain firing fields of different sizes. The neurons in the grid model, however, have regularly repeating firing fields, and the fields of one neuron (and of all neurons in a module) are all the same size. Note that we will not address whether the biological realization of an ideal grid code is feasible, plausible, or realistic. Rather, we will use the grid code as a lower bound on the accuracy that any spatial code will achieve.

### 2.2 Neuron model

All networks introduced in Section 2.1 have dynamics that are based on Eliav et al. (2021), briefly summarized as follows. According to Eliav et al. (2021), the synaptic current of a single neuron *i* is defined by


(2) $$\begin{array}{l} \tau \frac{dh_{i}}{dt}=-h_{i}+\underset{j}{\sum }W_{ij}g\left(h_{j}\right)+I_{bck}+I_{i}^{\text{pos}}\left(t\right), \end{array}$$

with τ being the time constant of the membrane and *I*_*bck*_ being a uniform background input (noise). *W*_*ij*_ defines the model-dependent connection strength between neuron *i* and neuron *j* based on their positional labels (bins) *x*_*i*_ and *x*_*j*_, respectively:


(3) $$\begin{array}{l} W_{ij}=W_{exc}e^{-\frac{\left|x_{i}-x_{j}\right|}{L_{int}}}-W_{inh} \end{array}$$

The interaction length *L*_*int*_ thereby determines the length over which neurons have positive connections and the weight constants *W*_*exc*_ and *W*_*inh*_ influence the amount of excitation and inhibition of this connection, respectively. The neuronal gain function *g*(*h*) is a threshold-linear function of the form


(4) $$g(h)=\{\begin{array}{ll} h & \text{if }h>0\\ 0 & \text{if }h<0 \end{array}$$

The positional input $I_{i}^{\text{pos}}$ defines the individual input each neuron receives based on the position of its fields and the respective distance of those to the current position of the agent


(5) $$\begin{array}{l} I_{i}^{\text{pos}}\left(t\right)=\underset{p}{\sum }I_{\text{pos}}e^{\frac{\left|x_{i}^{p}-\text{pos}\left(t\right)\right|}{L_{int}}}, \end{array}$$

where pos(*t*) defines the position of the agent at time *t* within the 1D environment, assuming a constant speed of 10m/s.

Beyond these general network dynamics, we introduced a variable, noisy background input, replacing *I*_bck_ in some experiments. The noisy background input is defined by a mean ($I_{\text{noise}}^{\mu }$) as well as a standard deviation ($I_{\text{noise}}^{\sigma }$) of the normal distribution generating the noisy input values.

### 2.3 Optimization

Biologically inspired *evolutionary optimization* is a prime candidate for finding the most suitable parameter configurations for the models defined above, as little prior knowledge is needed and few assumptions are required. Within this section, we briefly discuss how we used evolutionary optimization to find new parameter configurations that led to improved accuracy or energy efficiency in the models.

**Figure 3:**
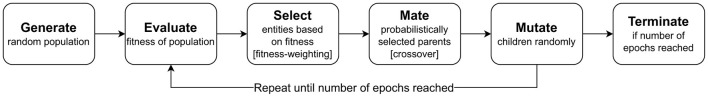
Flow diagram of the evolutionary optimization process.

The individual steps of our optimization algorithm are depicted in Figure 3 and are based on Simon (2013). We first *generate* a set of *N*_*pop*_ models (commonly *N*_*pop*_ = 20) for which selected network parameters are randomly initialized. Each parameter is subject to a lower as well as an upper bound, and parameter values are discretized to reduce the search space.

Then, the performance of all representative networks is *evaluated* using a *fitness function*, which we define below. To ensure reliable results, we commonly simulate 20 runs of the same network with different initial conditions, given that the positional accuracy of decoding can vary greatly for different field locations. The particular fitness function we use for this evaluation is based on the mean or median error of the network and is defined as


(6) $$f=e^{-E_{pos}^{\mu }*5/\frac{L_{env}}{N_{neu}}},$$

where$E_{pos}^{\tilde{\mu }}$ is the average or median ($E_{pos}^{\tilde{\mu }}$) of multiple mean positional decoding errors, calculated from several runs with the same network parameters, *L*_*env*_ is the total length of the environment in meters and *N*_*neu*_ is the total number of neurons. The constant 5 was simply introduced to scale the fitness function up.

Subsequently, a number of entities to keep for the next generation is *selected* from the entire population. This is done using fitness-weighting, i.e., the entities are ordered by their fitness first and then a subset of them is selected based on the defined selection rate *R*_*sel*_ (commonly *R*_*sel*_ = 0.2).

From this new set of entities, parents are chosen for *mating*, with a probability proportional to their fitness. Based on two chosen parents, a child entity is generated with parameters inherited from both parents. This inheritance is performed randomly. An integer is randomly generated, dividing the number of optimization parameters into two halves, one from each parent.

The optimization parameters of the children created in this step are then randomly *mutated* with a probability *P*_*mut*_ (commonly *P*_*mut*_ = 0.2). The parameters chosen to be mutated receive a new, randomly chosen value within the predefined boundaries of the respective parameter.

As a final step, a new population is created from the children. In all of our experiments, we additionally kept the entity with the best fitness from the selected entities constant without mating or mutating its parameters. This entire process is continued until the defined number of epochs, *EP*, is reached (commonly *EP* = 3, 000).

## 3 Experimental evaluation

The networks introduced in the previous section form the basis of our simulated experiments presented within this section. We first describe the general setup of the experiments. Then we introduce the results of the baseline models by Eliav et al. (2021), as well as their optimization with and without (*W*_*exc*_ = *W*_*inh*_ = 0) lateral connections. Evaluating networks without lateral connections allows us to analyze the usefulness of the MSMF code itself, i.e., decoupled from biological inspiration or plausibility, while analyzes of networks with lateral connections yield insights into the possible network structures that generate them.

### 3.1 Experimental setup and metrics

In order to rule out outliers, each experiment presented in this section with a single set of parameters was evaluated by performing 20 simulations of the same network with different initial conditions (i.e., random seed, leading to e.g., different field locations) and calculating the statistics (mean, median, standard deviation) of the positional error, the number of fields and other metrics. We commonly make use of the median, since the distribution of most metrics over the 20 runs is not Gaussian. All MF networks created and optimized within this section have a fixed number of *N*_*neu*_ = 50 neurons, except for one of the original models introduced by Eliav et al. (2021), which has *N*_*neu*_ = 4, 000 neurons. With this decision, we align our experiments with the theoretical evaluation performed by Eliav et al. (2021). These evaluations have demonstrated that 50 neurons are sufficient for accurately decoding the position in an environment of 200m. Nonetheless, we did perform some experiments with an increased number of neurons. The results, however, did not reveal significant differences, besides the obvious improvement of the decoding accuracy. For an evaluation of the performance of the original models (before optimization) with a varying number of neurons please see Supplementary Figure 1.

For some of the evaluations we also use an efficiency measurement as a comparison metric. We therefore define the median expected energy consumption for multiple runs of the same network as


(7) $$C_{eng}^{\tilde{\mu }}\;=\;N_{bins}\;*\;F_{all}^{\tilde{\mu }},$$

where *N*_*bins*_ is the total number of bins of the environment (for most experiments $N_{bins}=\frac{L_{env}}{L_{bin}}=\frac{200\text{m}}{0.5\text{m}}=400$) and $F_{all}^{\tilde{\mu }}$ is the mean in-field activity (firing-rate) of all fields (active as well as inactive).

The original models, based on the parameters by Eliav et al. (2021), as well as the ones generated using evolutionary optimization, will be abbreviated by *F/D/G-Org* and *F/D/G-Opt*, respectively. The first letter indicates the type of model, i.e., F-MF (*F*), D-MF (*D*), or grid (*G*). We indicate that a model contains lateral connections (*D-Org-1*^+^) or not (*D-Org-1*^−^), and also whether the connections in this model were optimized (*D-Org-1*^+*o*^) by the respective superscripts “+,” “−,” and “*o*.” In case the model receives a uniform background input (*I*_*bck*_), this is indicated by a subscript “β” (${\text{D-Org-1}}_{\beta }^{+}$).

While the goal of this evaluation is to find optimal configurations of MSMF networks, we note that evolutionary optimization does not guarantee that the multi-scale or multi-field properties are preserved; indeed, one or both properties could be lost in the course of optimization.

### 3.2 Original models

The first part of our evaluation consists of experiments performed with the original models and simulations introduced by Eliav et al. (2021). We evaluated both the F-MF and F-MF networks in order to analyze their positional encoding performance, answer the question of whether these networks are generally capable of reproducing the results of the theoretical analysis by Eliav et al. (2021), and identify potential ways to improve their performance.

**Figure 4:**
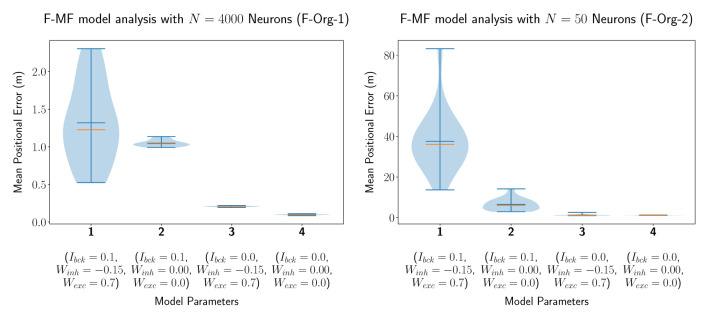
The distribution of the mean positional error of 20 individual runs of the original F-MF model with *N*_*neu*_ = 4, 000 neurons **(A)** as in the results from Eliav et al. (2021) and *N*_*neu*_ = 50 neurons **(B)** as in the theoretical analysis. The blue lines represent the minimum, maximum, and mean of the evaluation results, the orange line represents the median of it.

In our first experiment, we evaluated an F-MF model with identical parameters as proposed by Eliav et al. (2021), i.e., we simulated the network with a total number of *N*_*neu*_ = 4, 000 neurons. We then modified the parameters of the lateral connections in the network (*W*_*exc*_, *W*_*inh*_) as well as the noise or background input (*I*_*bck*_) in order to evaluate their impact on the encoding performance of the network. The statistics of the mean positional error for four models with different parameter combinations are visualized in Figure 4A. This simulation shows, that all three parameters have a significant influence on the accuracy of the network. Setting the background input as well as all lateral connections to zero results in a decrease of the median of the average positional error $E_{pos}^{\tilde{\mu }}$ by 1.128m (1.226m → 0.098m). Especially the background input has a significantly negative effect on the median performance (see models 3 and 4). The lateral connections, on the other hand, seem to have a strong influence on the standard deviation, leading to a broader overall distribution including both, networks with better as well as worse performances than without lateral connections. These results are further backed by the same experiment performed with only *N*_*neu*_ = 50 neurons, shown in Figure 4B, leading to similar results on a different scale (positional decoding error). The only remarkable difference compared to the experiments with *N*_*neu*_ = 4, 000 is the larger influence of *I*_*bck*_ on the mean/median of the distribution. The number of neurons was set to 50 here because this is the same number of neurons that is used by Eliav et al. (2021) in their theoretical evaluations. Both models are capable of encoding the agent's position with <2m decoding error, similar to the results by Eliav et al. *F-Org-1*, however, requires 4,000 neurons to achieve this result. *F-Org-2*, on the other hand, can only achieve this result without any background input.

**Figure 5:**
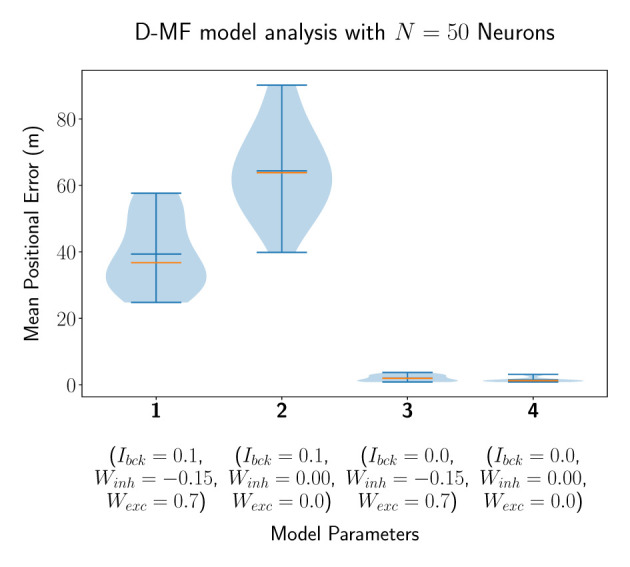
The distribution of the mean positional error of 20 individual runs of the D-MF model with *N* = 50 neurons. The blue lines represent the minimum, maximum, and mean of the evaluation results, the orange line represents the median of it.

In a second experiment, we evaluated the D-MF model, introduced in Section 2.1.2. The purpose of this experiment is to create a baseline comparison to the theoretical results by Eliav et al. and also evaluate the network in order to define further experiments for analyzing its properties and performance. We chose the connection parameter *TH*_*fsr*_ to be equal to 90% based on experimental results. The remaining parameters, such as for the gamma distribution of the field sizes, were chosen to be the same as for the theoretical analysis by Eliav et al. The results for *N*_*neu*_ = 50 neurons are visualized in Figure 5. Interestingly, the median of the average decoded positional error ($E_{pos}^{\tilde{\mu }}$) in this case is higher when the lateral connections are removed while the background input persists (model 1 vs. 2). This stands in contrast to the results obtained with the F-MF model and might be an indication, that these connections stabilize and denoise the system. Even when comparing the two last runs with each other, although the median and mean error are lower when all lateral connections are removed, the minimum error (of any of the 20 models) is even smaller for the third compared to the fourth model (0.858 vs. 0.866m). The implications of these insights on the significance of lateral connections in MF networks are further analyzed in Section 3.4.3. Similar to the *F-Org-2* results, the D-MF model is not capable of encoding the position with an error below 2m when background input is present.

The results presented in this section show that the MF networks are capable of reproducing the results from the theoretical analysis of Eliav et al.— but only under certain circumstances. The crucial factors that influence the positional encoding performance of these networks are the lateral connections and especially the noise (background input). In the remainder of this evaluation, we will, therefore, focus not only on the potential theoretical performance of MF networks but also on the (dis-)advantages of the lateral connections in such a multi-line attractor as well as the influence of different kinds of noise on the system. The goal of these analyzes is to answer the question of whether a system with such a code could be modeled by an attractor network and what the properties of this network are.

### 3.3 MSMF code

Within this part of the evaluation, we focus on the analysis of the MSMF code. Unless otherwise stated, the networks have no lateral connections nor do they receive background noise. In other words,


(8) $$\begin{array}{l} W_{exc}=W_{inh}=I_{bck}=0 \end{array}$$

for all models discussed in this section.

#### 3.3.1 Optimal parametrization of MF models

In the first deeper analysis of the MF models, we optimize only with respect to accuracy, seeking the best models that minimize the mean positional error of the network. Nevertheless, we will also compare their expected energy consumption, as defined by Equation 7. The configuration for the evolutionary optimization runs of the F-MF, as well as the D-MF models, is shown in Supplementary Table 1. A visualization of the optimization results can be found in the supplementary material in Supplementary Figures 5A, B, 6A, B, respectively. For the D-MF model, we ran multiple optimizations, continuously shifting the range of α, since the results kept improving. We included one row representing all runs—including the average number of generations of all runs.

The sampled parameter combinations for the F-MF model, shown in Supplementary Figure 5A, illustrate that, in general, a higher number of fields ($N_{f}^{\tilde{\mu }}$), i.e., more neurons per attractor (high *P*_*att*_), is preferable over lower numbers for achieving a low positional decoding error. This completely aligns with the results from the D-MF model, visualized in Supplementary Figures 6A, B. The networks achieving the highest decoding accuracy are all located in the range of θ <0.04. With θ this small, the average sampled field size also becomes very small and the number of fields therefore very large. This is specifically evident in Supplementary Figure 6B, where the number of fields is shown as a color for all networks with $E_{pos}^{\tilde{\mu }}\;<\;1.0$. All remaining networks maintain a large number of fields ($N_{f}^{\tilde{\mu }}\;>\;50$). The networks with the lowest decoding error from Supplementary Figure 6A also have the highest number of fields in Supplementary Figure 6B.

**Figure 6:**
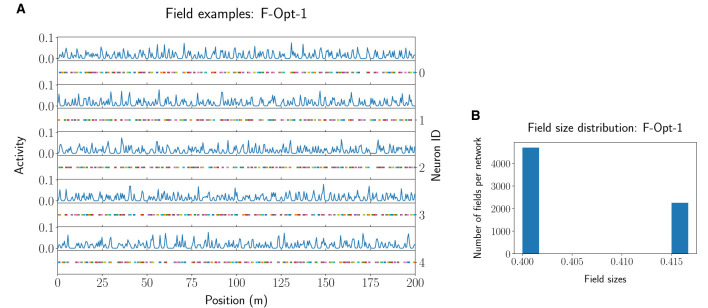
Example activity and fields for five neurons of the *F-Opt-1* network model **(A)**, together with the respective field size distribution **(B)**.

Further filtering of the values of the F-MF results (Supplementary Figure 5B) uncovers that, at least for this model, diverse parameter combinations can yield optimal networks with no positional decoding error ($E_{pos}^{\tilde{\mu }}\;=0.0$). We, therefore, included three different networks from the optimization results in Table 1. The first two networks achieve an optimal decoding error although the number of fields per neuron differs significantly for each of them. We picked *F-Opt-1* because it maintains the largest number of fields of all optimal network configurations ($N_{f}^{\tilde{\mu }}\;=\;140.6$) and *F-Opt-2* because it maintains the lowest number of fields ($N_{f}^{\tilde{\mu }}\;=\;44.8$) while still having somewhat different scales, i.e., differences between the number of attractors in each level (see Supplementary Figures 2A, B). The third network, *F-Opt-3*, was chosen for further analysis in the next parts of this section, as it maintains a low positional error ($E_{pos}^{\tilde{\mu }}\;=\;0.150$) with only 12 fields per neuron ($N_{f}^{\tilde{\mu }}\;=\;12.0$). Noticeably, both, *F-Opt-1* and *F-Opt-3*, fulfill the properties of an SSMF rather than an MSMF model. Specifically, the peaks of their field size distribution are rather close to each other (see Figure 6, Supplementary Figure 2), especially when compared to the original models (see Supplementary Figure 3). In contrast, the field size distribution of *F-Opt-2* maintains two separate peaks with one being at a field size twice as large as the other one. This model therefore fulfills the properties of an MSMF network as defined in Section 2, although it only maintains two different scales instead of three or more. The energy consumption of *F-Opt-2/3* is significantly lower than that of *F-Opt-1* since the fields of the neurons cover less space. While *F-Opt-3*'s energy consumption is slightly higher than *F-Opt-2*'s, it is significantly smaller than that of *F-Org-1*, showing that a better positional accuracy can be achieved with many small fields (140.6 vs. 2.4) instead of a high number of neurons (50 vs. 4, 000), while also reducing the energy consumption.

**Table 1:** Optimized F-MF models without lateral connections.

**Model ID**	***N*_*A*_*L*__0__**	***N*_*A*_*L*__1__**	***N*_*A*_*L*__2__**	***Patt***	***N*_*neu*_**	** $N\tilde{\mu }f$ **	** $C\tilde{\mu }eng$ **	** $E\tilde{\mu }pos$ **	***Eminpos***	***Emaxpos***
F-Opt-1	50	48	50	0.95	50	140.6	140.8	0.000	0.000	0.003
F-Opt-2	50	22	40	0.40	50	44.8	60.0	0.000	0.000	0.004
F-Opt-3	11	10	9	0.40	50	12.0	59.6	0.150	0.133	0.279
F-Org-1	5	2	1	0.30	4,000	2.4	3,460.6	0.098	0.085	0.110
F-Org-2	5	2	1	0.30	50	2.4	43.8	1.148	1.056	1.293

This finding is confirmed by the results of the D-MF model. As stated before, the optimization results favor field sizes that are tightly distributed around a small mean value. Quantitatively, for all evaluated networks with a median error $E_{pos}^{\tilde{\mu }}\;<\;1.0$, the median of the distribution of all field size means is 0.41, the median of the variance is 0.01 (see Supplementary Figure 4). Due to these resultant properties, the models cannot properly be defined as multi-scale models anymore. Furthermore, their accuracy is inferior to that of the F-MF models. Table 2 shows that the energy consumption ($C_{eng}^{\tilde{\mu }}$) of the D-MF models is much higher for both the optimal as well as the original model when compared to the energy consumption of the *F-Opt* models (89.5>> 44.8), while the median of the positional decoding error is much higher than that of the most optimal F-MF models (*F-Opt-1/2*). Since the optimized F-MF models are now either (a) no longer multi-scale models or (b) maintain fewer scales than the original models, the primary difference between the D-MF and the F-MF models lies in the accurate, evenly distributed placement of the fields in the F-MF networks compared to the D-MF networks; the even spacing might have a larger effect on the decoding accuracy than the multi-scale property of the field distributions.

**Table 2:** Optimized D-MF models without lateral connections.

**Model ID**	**α**	**θ**	** $\bar{\Sigma }fs$ **	***N*_*neu*_**	** $N\tilde{\mu }f$ **	** $C\tilde{\mu }eng$ **	** $E\tilde{\mu }pos$ **	***Eminpos***	***Emaxpos***
D-Opt-1	15.92	0.02	36	50	114	89.5	0.300	0.009	1.580
D-Org-1	3.16	1.80	30	50	7.13	74.2	1.265	0.866	3.141

In addition to these findings, many models, but especially the D-MF models, had a high variance in the decoding error across different runs with the same parameters but varying initialization of field locations and sizes. For both models, *D-Org* and *D-Opt-1*, the discrepancy between the minimum of all mean decoding errors of 20 runs and the maximum is significant [$\Delta E_{pos}^{\tilde{\mu }}(\text{D-Org})=2.275$, $\Delta E_{pos}^{\tilde{\mu }}(\text{D-Opt-1})=1.571]$. Since it occurs for both models almost at an equal level, the shape of the gamma distribution as well as the number of fields do not seem to be determining factors.

**Figure 7:**
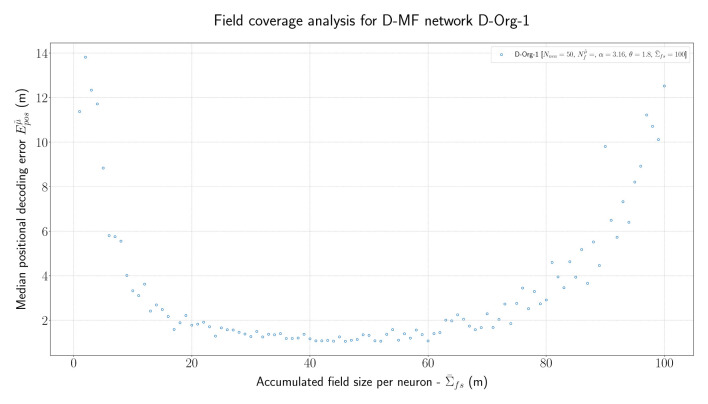
The median positional error for a range of experiments performed with the *D-Org-1* model, varying the maximal field coverage (${\bar{\Sigma }}_{fs}$ ∈ {1,2,...,100}m).

In order to further investigate the optimal parametrization of the networks, we analyzed the influence of the maximal field coverage of a neuron (${\bar{\Sigma }}_{fs}$). For this experiment, we ran the original D-MF model ( *D-Org-1*) and varied the value for ${\bar{\Sigma }}_{fs}$ between each run in a range from 1 to 100. The median of the resulting positional error is visualized in Figure 7. The results are twofold. First, the mean/median measured experimentally (30m) by Eliav et al. (2021) lies within the minimum of this plot, which corroborates the parameter and model choice. In the experiments, however, many cells had much larger field coverage; in fact, a significant number had a field coverage ${\bar{\Sigma }}_{fs}$ > 100 m. With the given parameters, such field sizes would lead to a significant drop in the positional decoding accuracy (>10m) compared to the accuracy achieved with optimal parameter values at around ${\bar{\Sigma }}_{fs}$ = 40m. Alternatively, either this model, or at least its parameters, are not suited for representing the given MSMF code, or the given MSMF code is not just a “simple” place code.

#### 3.3.2 D-MF variation analysis

In the previous section, we optimized the parameters for different MF networks. The experiments demonstrated that the performance of these networks is highly unstable, i.e., the same parametrization does not necessarily lead to the same or even a similar accuracy on different runs. We now investigate extreme scenarios in which a network with the same parameters produces a large and a small error when initialized differently. The goal of this evaluation is to identify possible factors of the place field distribution that have either a beneficial or detrimental effect on the decoding accuracy. For instance, we ask whether a distribution close to uniform, hence similar to a grid code, is beneficial, or whether a high number of falsely active cells leads to errors in the decoding.

In order to address these questions, we compared the results of the *D-Opt-1* and the *D-Org-1* model (see Table 2). Both networks have a high variation between the minimum and maximum mean positional decoding error, depending on the field initialization but with the same parameters. They do, however, differ significantly in their field size distribution; model *D-Opt-1* has a large number of fields ($N_{f}^{\tilde{\mu }}$ = 114) while model *D-Org-1* has a low number of fields ($N_{f}^{\tilde{\mu }}$ = 7.13).

The analysis we conducted in order to identify possible problems with these networks include:

- The percentage of unique field combinations,
- The average number of false positive/negative bins,
- The average distance between all field locations and the nearest bin location (centers),
- The divergence of field size/location distribution from their respective actual distribution.

The results of these analyzes are visualized in Supplementary Figure 7. They do not indicate that there is a pattern, convergence, or correlation between the decoded positional error $E_{pos}^{\tilde{\mu }}$ of a network and any of the aforementioned properties. The high divergence in positional decoding accuracy between different runs must therefore result from the randomness of the field locations. We could not find or verify any other explanation for this phenomenon.

#### 3.3.3 Benchmark against the grid code

In order to put the results from the original and optimized MF models into context, we compare them in this section to the results from multiple optimized one-dimensional grid codes. Each code is built by a network with multiple modules (*N*_*mod*_), each of which contains a certain number of neurons ($N_{neu}^{mod}$). The modules have different scales, with a minimum scale ($S_{mod}^{min}$) and a multiplier from one scale to the next (*S*_*mod*_). All these parameters were optimized over 3000 epochs without any lateral connections or background input (*W*_*inh*_ = *W*_*exc*_ = *I*_*bck*_ = 0). The results of a few exemplary networks that minimize the positional decoding error but nevertheless have different properties are listed in Table 3.

**Table 3:** Optimized grid models without lateral connections.

**Model ID**	***N*_*mod*_**	** $N_{neu}^{mod}$ **	***S*_*mod*_**	** $S_{min}^{min}$ **	** $N\tilde{\mu }f$ **	** $C\tilde{\mu }eng$ **	** $E\tilde{\mu }pos$ **	***Eminpos***	***Emaxpos***
G-Opt-1	3	9	1.6	0.5	29.852	30.75	0.0	0.0	0.0
G-Opt-2	9	19	3.0	0.5	3.509	64.57	0.0	0.0	0.0
G-Opt-3	9	7	3.0	0.5	9.524	64.64	0.0	0.0	0.0
G-Opt-4	3	19	1.2	0.5	17.737	30.76	0.0	0.0	0.0
G-Opt-5	3	19	1.8	0.5	13.07	30.73	0.0	0.0	0.0

The optimization of the grid code shows that with at least three modules and four neurons per module, almost all combinations of the grid model achieve the same or even better positional decoding accuracy as the best optimized MF models introduced so far. We picked five samples from the optimized models, each with a different number of modules, neurons per module, and module scale, all of them achieving a perfect median decoding error of $E_{pos}^{\tilde{\mu }}$ = 50. The networks can be categorized as follows:

G-Opt-1: Lowest number of neurons overall (27).
G-Opt-2: Largest number of neurons overall (171).
G-Opt-3: Large number of modules, small number of neurons.
G-Opt-4: Large number of neurons, small number of modules.
G-Opt-5: Same as *G-Opt-4* but with a much larger module scale.

The reason why we picked these models is to evaluate the performance of different combinations of module size, number of neurons, and module scale. In the evaluation results focusing on the positional decoding error and energy consumption, shown in Table 3, there are no differences in the accuracy between the networks. The energy consumption, on the other hand, increases significantly when the number of modules rises. This can be expected since each new module adds another layer of $N_{neu}^{mod}$ neurons, resulting in additional activity and hence increased energy consumption.

**Figure 8:**
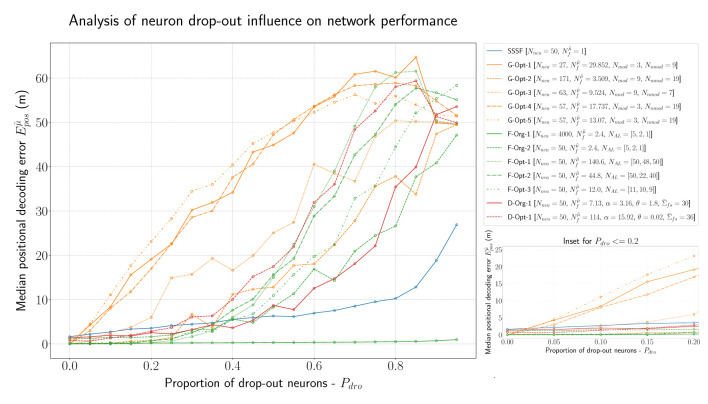
Evaluation of all introduced models (F-MF, D-MF, Grid, and SSSF) with an increasing percentage of drop-out neurons (*P*_*dro*_∈{0.0, 0.95, 0.05}). An inset of the same data with *P*_*dro*_ ≤ 0.2 **(right)**.

In order to analyze the robustness of all models described so far in this evaluation, we conducted further experiments with a certain percentage of drop-out neurons. Figure 8 visualizes the results of this experiment. By far the best-performing model is, as expected, the *F-Org-1* with 4,000 neurons overall. Even in the worst case, with 95% of the neurons being dead, it still performs better than most networks with just 5% lesions. All of the optimized F-MF models (*F-Opt-1/2/3*) are capable of maintaining a median positional decoding error $E_{pos}^{\tilde{\mu }}$ < 1m, even with a drop-out rate of *P*_*dro*_ = 0.25, i.e., 25% randomly removed neurons. This demonstrates the effect of the redundancy in these models, caused by the large number of fields per neuron. In particular, the redundancy in the *F-Opt-2/3* models makes them more robust than the grid code while maintaining a lower energy consumption than the best-performing grid model, *G-Opt-2*. Almost all grid models perform significantly worse than the other models, even when only 5% of the neurons are disabled. Only the *G-Opt-2* model performs comparably well to the optimized F-MF models. It does, on the other hand, require a significantly larger number of neurons for that to occur ($N_{neu}^{mod}=171$). This shows, that in order to gain robustness in grid models, one needs a large number of modules and neurons to achieve redundancy.

### 3.4 Lateral connections in MF models

The last part of our evaluation focuses on the lateral connections in the MF models, i.e., the connections that are essential for making it a CAN.

#### 3.4.1 Optimized MF models with lateral connections

**Figure 9:**
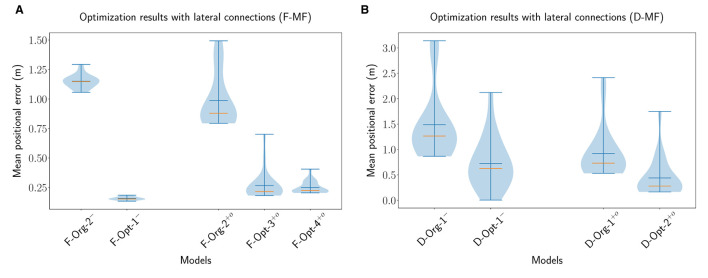
The distribution of the mean positional error of 20 individual runs for optimized F-MF **(A)** and D-MF **(B)** models with lateral connections. On the left of each figure the results from the previous experiments without lateral connections are shown. On the right, the results from the optimization are visualized. The blue lines represent the minimum, maximum, and mean of the evaluation results, the orange line represents the median of it.

For the proper evaluation of the purpose or benefits of the lateral connections in the MF models, we performed multiple optimizations of the models with different parameterizations. For each network (D-MF, F-MF) we performed three optimizations: the first one optimizes for all parameters of the network (lateral connections and architecture/field distribution), resulting in a new model; the other two optimize the lateral connection parameters of existing models (e.g., *F-Org-1*) while the architecture and field distribution remain constant (applied to original and optimal models). The parameters for training the networks are listed in Supplementary Table 2, the trained parameters of the networks are listed in Supplementary Tables 3, 4. The optimization results are visualized in Figure 9.

For the F-MF model, we optimized the lateral connection parameters of the *F-Org-2* and *F-Opt-3* models, resulting in the *F*−*Org*−1^+*o*^ and *F*−*Opt*−3^+*o*^ models, respectively. An initial optimization of the *F-Opt-1/2* models with lateral connections resulted in positional decoding errors far too high for further experiments, even after several hundred epochs of training. We therefore continued with the *F-Opt-3* model, as it led to a reasonably low positional decoding error with optimized lateral connections. In addition to that we optimized all parameters, including the architectural parameters, resulting in the new model *F*−*Opt*−4^+*o*^. We kept the maximum number of attractors per level quite low in this case, due to the aforementioned issue with training lateral connection weights for models with a large number of attractors (*N*_*AL*_>>30).

The evaluations of these networks (Figure 9A) show, that the lateral connections reduce the median decoding error for the original network architecture (*F*−*Org*−2^−^ vs. *F*−*Org*−2^+*o*^) and increase it for the optimized architecture (*F*−*Opt*−3^−^ vs. *F*−*Opt*−3^+*o*^/*F*−*Opt*−4^+*o*^). This indicates that lateral connections are more beneficial in a spatial code with fewer but larger fields per neuron since the *F-Opt-3* model has significantly more fields per neuron than the *F-Org-2* model [$N_{\tilde{\mu }}^{f}\left(\text{F-Opt-3}\right)=12.0$ vs. $N_{\tilde{\mu }}^{f}\left(\text{F-Org-2}\right)=2.4$, cmp. Table 1].

We performed the same three optimizations for the D-MF model. The results shown in Figure 9B do not depict the results for the optimization of the *D-Opt-1* model, however. The reason for this is that this optimization did not lead to any results. After running it for 200 generations, the median decoding error was still around 50m. We, therefore, omitted this result and included the newly trained model *D*−*Opt*−2^+*o*^ instead. For this model, all parameters, including the lateral connections, were trained from scratch. This also resulted in the model *D*−*Opt*−3^+*o*^, which is less accurate but has a lower field size ratio threshold and will hence be used in later evaluations.

The results from the optimized D-MF networks confirm the indications that the analysis of the F-MF optimizations already revealed—especially networks with fewer and larger fields benefit from lateral connections. This seems intuitive since more fields also lead to more connections and with that to more noise. Creating only a few connections with small weights, however, seems to stabilize the system and reduce noise. In addition to that, we observed that most of the weights of the optimized models were in fact negative, for some of them even all weights. This applied especially to the cases where the decoding error dropped by introducing the optimized weights. We will analyze the influence of the weights on the firing fields of individual neurons further in Section 3.4.3.

**Figure 10:**
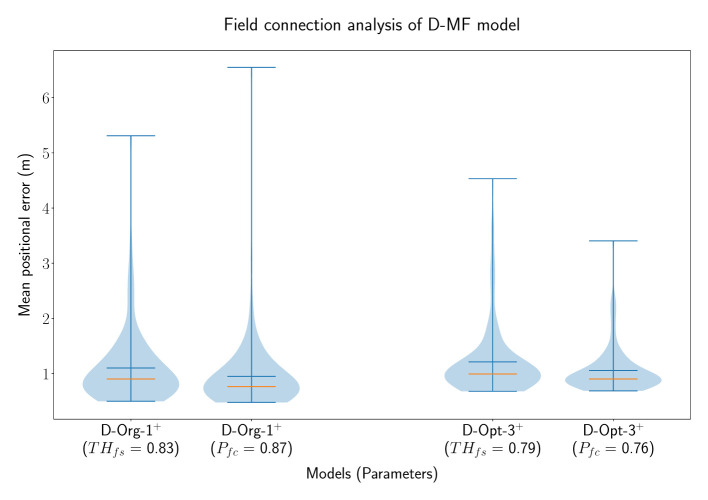
The distribution of the mean positional error of 100 individual runs of pairs of D-MF models, with either the field ratio threshold or an equivalent field connection probability set. The blue lines represent the minimum, maximum, and mean of the evaluation results, the orange line represents the median of it.

In order to analyze the general benefit of connecting two neurons based on the individual field sizes of the neurons, we performed an additional experiment with D-MF models only. In this experiment, two different models were chosen from the optimization results, *D*−*Org*−1^+*o*^ and *D*−*Opt*−3^+*o*^. The latter one resulted from the same optimization as *D*−*Opt*−2^+*o*^. We decided to use the given model for this evaluation due to its more relevant field size ratio threshold (*TH*_*fsr*_ = 0.79) for this experiment, compared to the more accurate model, *D*−*Opt*−2^+*o*^, used before (*TH*_*fsr*_ = 0.99). Both models were evaluated 100 times, one time with a field ratio threshold [$TH_{fsr}\left({\text{D-Org-1}}^{+}\right)=0.83$ and $TH_{fsr}\left({\text{D-Opt-3}}^{+}\right)=0.79$] and another time with a field connection probability [$P_{fc}\left({\text{D-Org-1}}^{+}\right)=0.87$ and $P_{fc}\left({\text{D-Opt-3}}^{+}\right)=0.76$]. The results of these experiments are visualized in Figure 10. These results indicate that there is no benefit in creating connections between neurons based on their respective field sizes. Creating random connections leads to very similar, but in both cases even smaller decoding errors. While we do not have an explanation for the decrease in the decoding error, we observed, that the fields of the networks with a field connection probability were sharpened equivalently to the sharpening which occurs when using a field ratio threshold. This property is further investigated in Section 3.4.3.

#### 3.4.2 CAN features in recurrent MF models

One of the key features of CANs is the maintenance of a bump of activity in the absence of a specific input. Some networks are capable of maintaining a bump of activity after the specific input is removed without receiving any input at all, while others need a certain amount of unified background input, all depending on the setup of the connections between neurons. In this part of the evaluation, we have looked at both of these cases to evaluate whether the MF models, particularly the original MSMF models, can achieve this and are indeed Continuous Attractor Networks or not. For this purpose, we create a baseline with an SSSF model with *N*_*neu*_ = 50 neurons spanning uniformly over the entire environment. We then remove the input for a length of *L*_*rem*_ = 20m and evaluate the network with and without lateral connections. If the lateral connections do create a CAN, then the decoding error is expected to be smaller with lateral connections present. During the time, where the positional input is removed, the optimal decoded position is standing still, i.e., it is equal to the last position where the positional input was active. This leads to a scenario, where the maintenance of a bump at the last known location after the positional input is removed results in an optimal decoded position. In this scenario, the lateral connections are essential to drive recurrent excitation in the network to maintain a bump of activity at the last input location. Without recurrent excitation, the activity would simply decline until the network activity vanishes.

**Figure 11:**
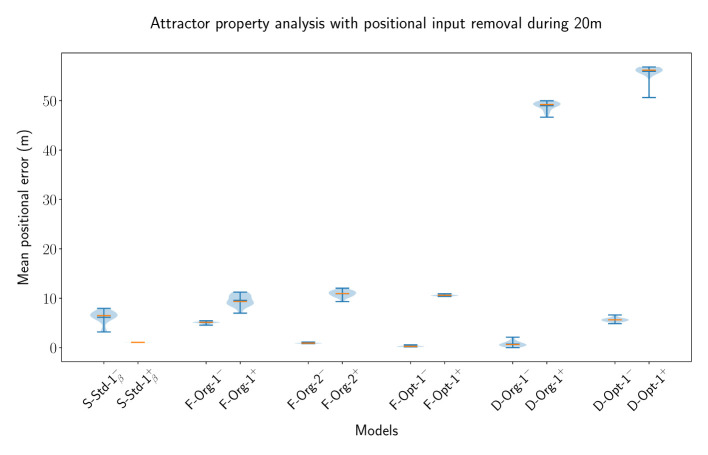
The error distribution of the decoded position for 20 individual runs of pairs of SSSF, F-MF, and D-MF models (without/with lateral connections). In this experiment, the position-dependent input signal is removed for 20m (10% of the entire length). The blue lines represent the minimum, maximum, and median of the evaluation results, the orange line represents the mean of it.

We picked multiple different models from the previous experiments and optimizations in order to verify, that the results are not based on a certain parametrization of the networks. For the F-MF model, we chose the *F-Opt-3* as a reference, since we could not successfully optimize any other network with lateral connections (see Section 3.4.1). The decoded error for all experiments is shown in Figure 11. The models are visualized pairwise, without and subsequently with lateral connections. If the respective model is a CAN, then the error should decrease from the first to the second run, as it is the case for the SSSF model (*S-Std-1*). This does, however, not apply for any of the MF models, including the multi-scale models from Eliav et al. (2021). On the contrary, the error increases significantly for all of the MF models. These results therefore show no evidence that the given MSMF and SSMF models are indeed CANs.

#### 3.4.3 Benefits of lateral connections in MF models

In the previous part of the evaluation, we have shown that the MF models do not seem to fulfill some typical properties of a CAN. In this final part of the evaluation we now investigate what other benefits or purposes the lateral connections could have in such a model. We therefore analyze the influence of the lateral connections on the field shape of the individual neurons in both, the F- and D-MF networks, by comparing models without lateral connections (“−”) to ones with optimized lateral connections (“+*o*”).

**Figure 12:**
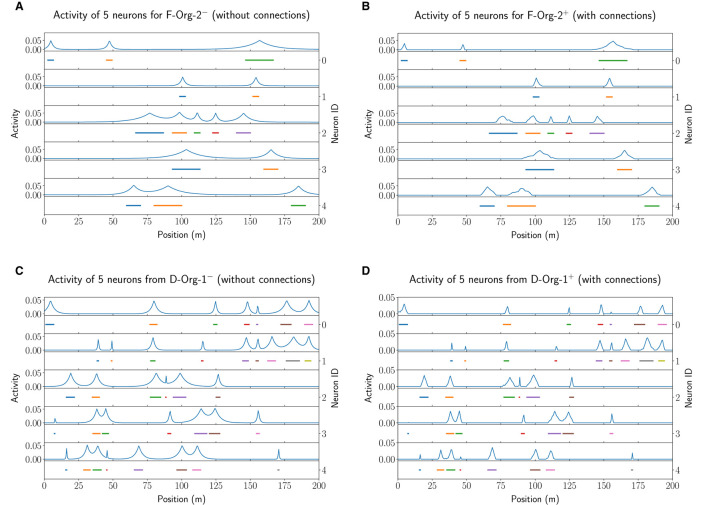
The field activity for the first five neurons of the first network of the experiment performed with 20 instances of *F*−*Org*−2^−^ **(A)** and *F*−*Org*−2^+^ **(B)** as well as *D*−*Org*−1^−^ **(C)** and *D*−*Org*−1^+^ **(D)**.

The results of this analysis are visualized in Figure 12 for the *F*−*Org*−2^−/+*o*^ (top row) and *D*−*Org*−1^−/+*o*^ (bottom row) models. In both cases, the activation of the lateral connections leads to a sharpening of almost all firing fields. Due to this sharpening the fields have less activity outside of their actual field and hence lead to less noise in the decoding (false positives). In Section 3.4.1 we already demonstrated that the lateral connections lead to a decrease of the positional decoding error in both optimized original models (*F*−*Org*−2^+*o*^ and *D*−*Org*−1^+*o*^). While this does not apply to all of the models, we do think that lateral connections in such an MF model could be used for de-noising the input data. This, however, seems to require few connections with small negative weights.

## 4 Conclusion

Training several networks using evolutionary optimization and comparing the resulting MF networks to an SSSF network (line attractor) as well as a grid code produced two main results that shed light on the accuracy and robustness of the multi-scale, multi-field place code recently found in the hippocampus of bats (Eliav et al., 2021).

First, we identified that both the SSMF and MSMF models outperform an SSSF code; the SSSF code reflects the classical concept of a hippocampal place code. Using evolutionary optimization, both SSMF and MSMF models can result from training MF networks. Yet, neither SSMF nor MSMF networks achieved the decoding accuracy of multi-scale grid codes. The reason is that a grid code's fields are optimally distributed for environments of any dimension (Mathis et al., 2015). In contrast, here we randomized the field locations for the (MS)MF networks; hence, these locations were not optimized. Our experiments on (MS)MF codes also showed that the decoding accuracy depends strongly on the specific instance of how random fields were placed, even given the same parameters for field generation. Due to the much larger number of fields in many of the MF models, however, these models are much more robust to noise induced by drop-out or lesions than grid codes, which have less redundancy.

Second, while the observed firing fields in bats were thought to be associated with multiple intermingled line-attractors (Eliav et al., 2021), we showed that they do not have one of the properties characteristic of continuous attractors. Specifically, when removing the position-dependent input for a short period of time, the networks would always converge to a single baseline attractor state, independently of the animal's current location. While this discrete attractor is always active in the background during the movement of the agent/animal, it is overridden by the location-specific input to the network, yet this input leaves no “memory” imprint. Instead, the primary benefit of the lateral connections that we could identify in these networks was the introduction of inhibition. This inhibition trims the “foothills” of the activity bumps, thereby creating more precise firing fields.

We note that the optimized models noticeably differed from the biological MSMF results of bats presented by Eliav et al. (2021). Specifically, the optimized models tend to lose the multi-scale property, resulting in narrow distributions of place field sizes in each neuron, while the number of fields is higher than what is observed in the experimental data [e.g., compare Figure 6B, Supplementary Figures 2B, D, 3B, D with Supplementary Figures 12, 13 of Eliav et al. (2021)]. Instead, the resulting models are close to an SSMF code, consistent with the results for rodents that were running on long linear tracks (cf. Rich et al., 2014, Figure 2). The difference in the structure of fields across different species, at least in long, linear environments, is not explained by our optimization results. Whether other factors play a role for bats remains an open question for future work.

Based on our results, we conclude that the MSMF place code found in the hippocampus of bats is unlikely to be the most suitable representation for space with respect to accuracy and energy efficiency, unless robustness to noise is also considered. Surprisingly, we found that the (MS)MF networks we investigated did not have continuous attractors. It is therefore possible that the bats' MSMF code does not directly inherit the continuous attractor network topology inherent in the head-direction system of mammals (Peyrache et al., 2015) and insects (Kim et al., 2017), which serves as an input stage to neuronal representations of space.

## Data availability statement

The datasets presented in this study can be found in online repositories. The names of the repository/repositories and accession number(s) can be found at: https://github.com/dietriro/msmf-code.

## Author contributions

RD: Investigation, Methodology, Software, Writing – original draft, Writing – review & editing, Conceptualization, Formal analysis, Validation, Visualization. NW: Conceptualization, Supervision, Validation, Writing – review & editing. MS: Conceptualization, Supervision, Validation, Writing – review & editing. AK: Funding acquisition, Project administration, Supervision, Writing – review & editing.

## Footnotes

^1^The source code for the experiments and reproduction of the figures is provided online at: https://github.com/dietriro/msmf-code.
