# Supplementary material for: Grid codes vs. multi-scale, multi-field place codes for space
Source: Front Comput Neurosci. 2024 Apr 19;18:1276292. doi: 10.3389/fncom.2024.1276292 (PMC11066179; doi:10.3389/fncom.2024.1276292)
Supplement: Supplementary file 1 [file Data_Sheet_1.PDF]

frontiers

Supplementary Material

Grid Codes versus Multi-Scale, Multi-Field Place Codes for Space

1 SUPPLEMENTARY MATERIALS

1.1 Evaluation

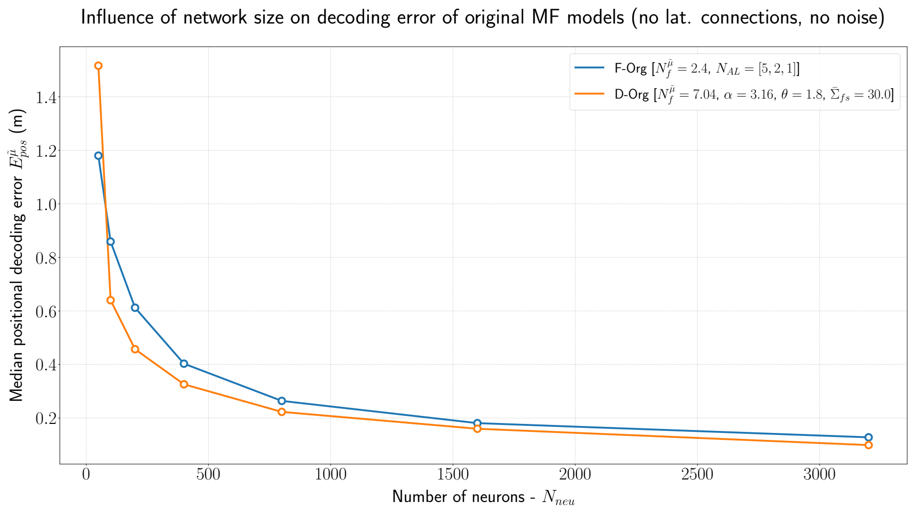

Figure S1a

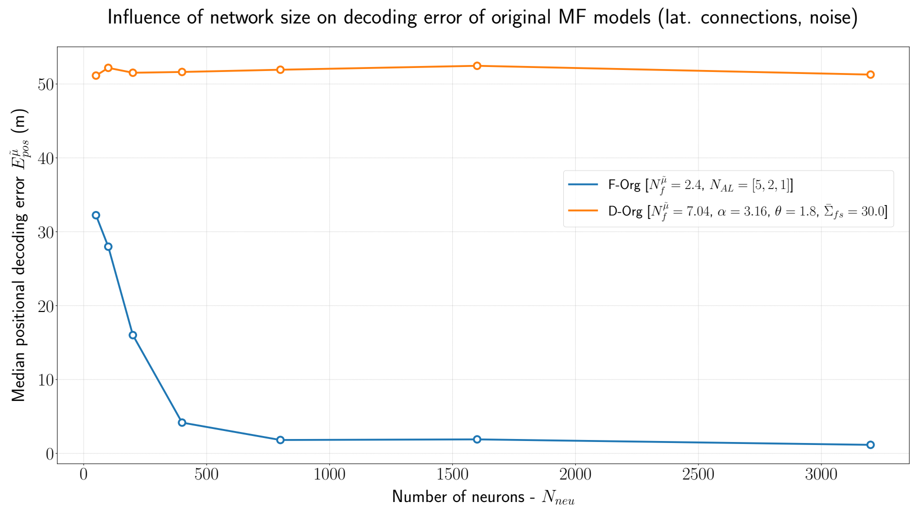

Figure S1b

Figure S1. The influence of the network size, i.e. number of neurons N_{neu}, on the positional decoding accuracy for the original networks of the F-MF and D-MF model shown without (a) and with (b) lateral connections and noise.

1.1.1 Optimal Parameterization of MF Models

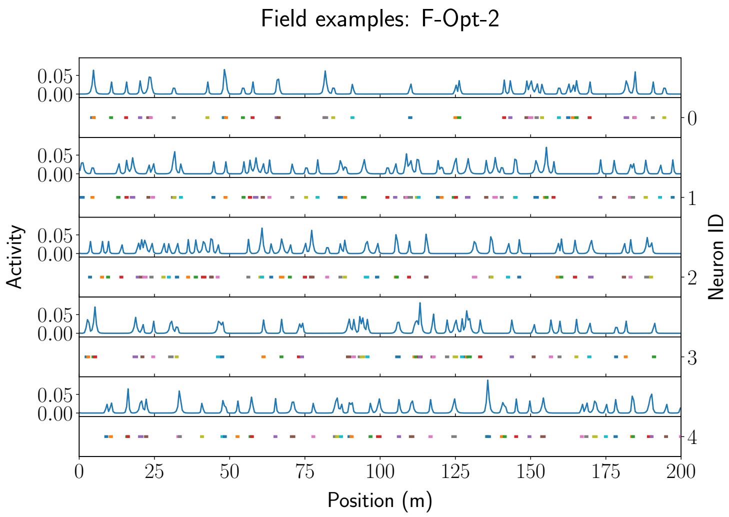

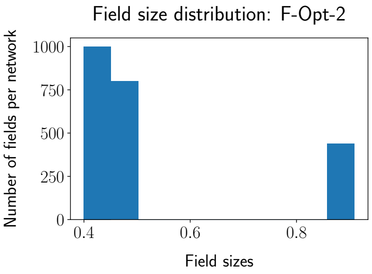

Figure S2a

Figure S2b

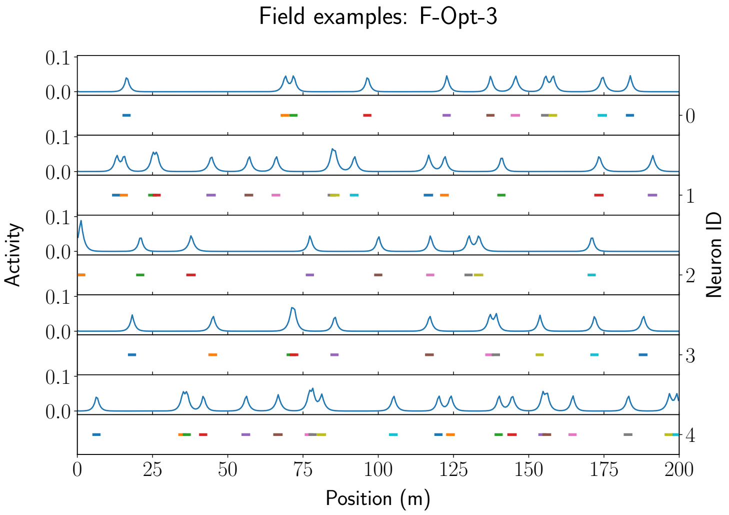

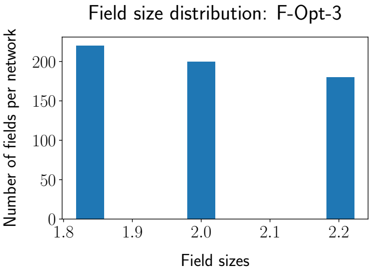

Figure S2c

Figure S2d

Figure S2. Example activity and fields for 5 neurons of the *F-Opt-2* and *F-Opt-3* network model (left column). The field size distribution for the respective network (right column).

Table S1. Parameters for optimization of F-/D-MF models without lateral connections.

Netw. Type	#Gen.	Param 1	Param 2	Param 3	Param 4
F-MF	3000	$N_{AL_0} \in \{1, 50, 1\}$	$N_{AL_1} \in \{1, 50, 1\}$	$N_{AL_2} \in \{1, 50, 1\}$	$P_{att} \in \{0.05, 1.0, 0.05\}$
D-MF	1610	$\alpha \in \{0.02, 30.00, 0.02\}$	$\theta \in \{0.02, 6.00, 0.02\}$	$\bar{\Sigma}_{fs} \in \{2, 100, 1\}$	

Table S2. Parameters for optimization of F-/D-MF models with lateral connections.

Netw. Type	Param 1	Param 2	Param 3	Param 4	Param 5	Param 6
F-MF	N_{AL_0} $\in \{1, 11, 1\}$	N_{AL_1} $\in \{1, 11, 1\}$	N_{AL_2} $\in \{1, 11, 1\}$	P_{att} $\in \{0.1, 0.5, 0.05\}$	W_{exc} $\in \{0.02, 1.6, 0.02\}$	W_{inh} $\in \{-0.08, -0.02, 0.02\}$
D-MF	α $\in \{0.02, 30.00, 0.02\}$	θ $\in \{0.02, 6.00, 0.02\}$	$\bar{\Sigma}_{fs}$ $\in \{2, 100, 1\}$		W_{exc} $\in \{0.02, 1.6, 0.02\}$	W_{inh} $\in \{-0.08, -0.02, 0.02\}$

Table S3. Optimized F-MF models with lateral connections. Only bold values were trained.

Model ID	W_{inh}	W_{exc}	I_{bck}	N_{AL_0}	N_{AL_1}	N_{AL_2}	P_{att}	$N_f^{\tilde{\mu}}$	$C_{eng}^{\tilde{\mu}}$	$E_{pos}^{\tilde{\mu}}$	E_{pos}^{min}	E_{pos}^{max}
F-Org-2^{+o}	**-0.1**	**0.04**	0.0	5	2	1	0.3	2.4	27.08	0.880	0.794	1.493
F-Opt-1^{+o}	**-0.04**	**0.2**	0.0	11	10	9	0.4	12.0	25.46	0.215	0.182	0.701
F-Opt-4^{+o}	**-0.04**	**0.22**	0.0	**11**	**10**	**9**	**0.45**	13.5	27.079	0.224	0.204	0.406

Table S4. Optimized D-MF models with lateral connections. Only bold values were trained.

Model ID	W_{inh}	W_{exc}	I_{bck}	TH_{fsr}	α	θ	$\bar{\Sigma}_{fs}$	$N_f^{\tilde{\mu}}$	$C_{eng}^{\tilde{\mu}}$	$E_{pos}^{\tilde{\mu}}$	E_{pos}^{min}	E_{pos}^{max}
D-Org-1^{+o}	**-0.04**	**0.08**	0.0	**0.83**	3.16	1.8	30	7.03	27.609	0.733	0.531	2.415
D-Opt-2^{+o}	**-0.04**	**0.58**	0.0	**0.99**	**2.7**	**0.72**	**40**	23.35	41.042	0.279	0.164	1.748
D-Opt-3^{+o}	**-0.04**	**0.26**	0.0	**0.79**	**5.4**	**1.74**	**51**	6.35	32.647	0.992	0.679	4.532

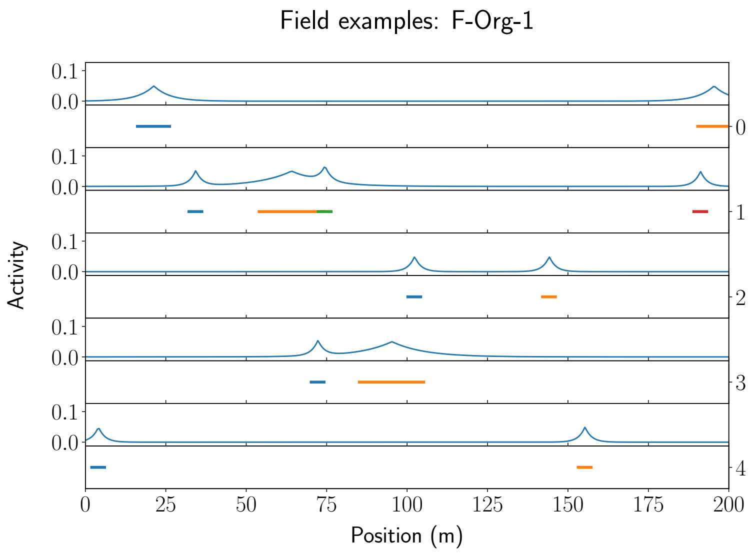

Figure S3a

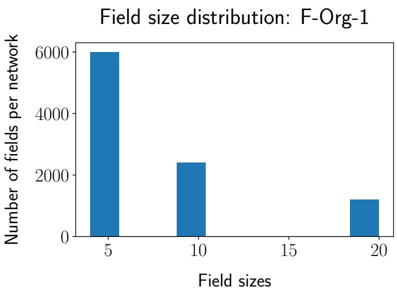

Figure S3b

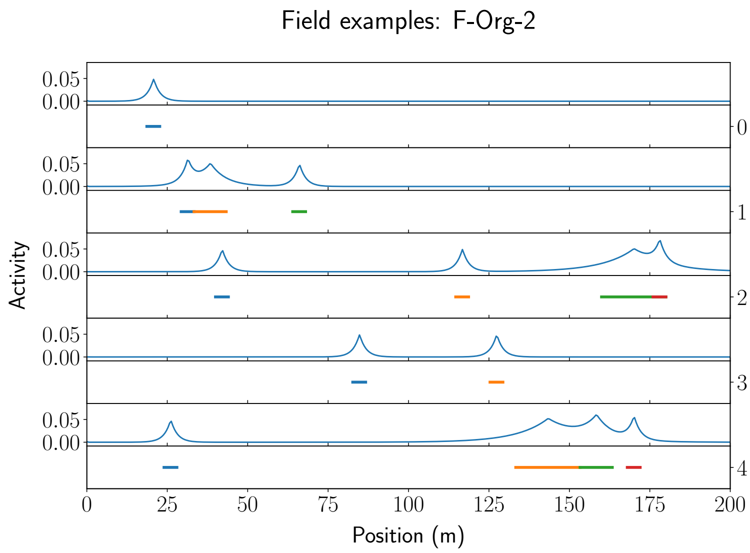

Figure S3c

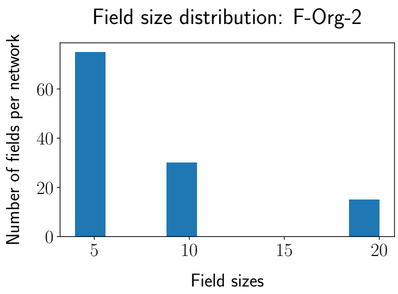

Figure S3d

Figure S3. Example activity and fields for 5 neurons of the *F-Org-1*/*F-Org-2* network model (left column). The field size distribution for the respective network (right column).

1.1.2 Lateral Connections in MF Models

1.1.3 D-MF Field Size/Location Distribution

In the previous section, we optimized the MF models in order to identify optimal parameter combinations. This experiment also demonstrated, that these networks are highly unstable, i.e. the same parameterization does not necessarily lead to the same or even a similar decoded positional error. Within this part of our evaluation we therefore evaluate specifically these extreme scenarios, in which a network with the same parameters produces a large and a small error when initialized differently. The goal of this evaluation is to

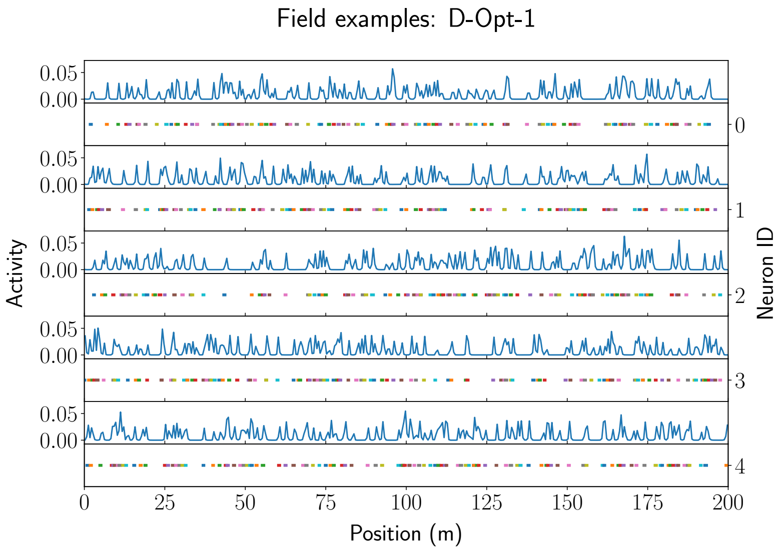

Figure S4a

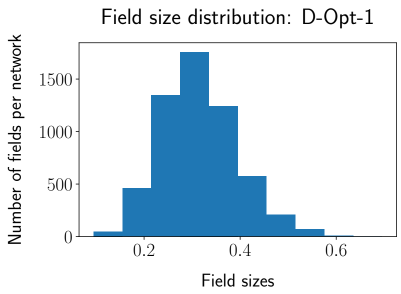

Figure S4b

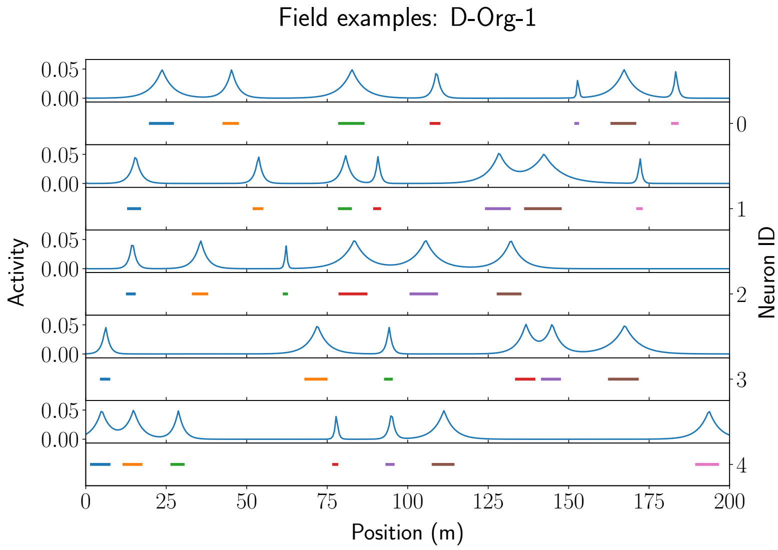

Figure S4c

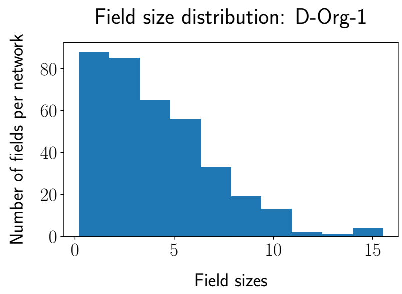

Figure S4d

Figure S4. Example activity and fields for 5 neurons of the *D-Opt-1*/*D-Org-1* network model (left column). The field size distribution for the respective network (right column).

identify possible factors of place field distribution which have a (non-)benefitial impact on the decoding accuracy, such as a uniform distribution similar to the grid code.

In order to evaluate this, we picked two models from the optimization results. Both networks have a high variation between the minimum and maximum mean positional decoding error which can be reached with the same parameters and different field initialization. We chose one model (A) with a large number of fields N_f^μ (high $\bar{\Sigma}_{fs}$) and another model (B) with a low number of fields N_f^μ (low $\bar{\Sigma}_{fs}$). The parameters for both models are shown in Table S5 together with some evaluation results on population level.

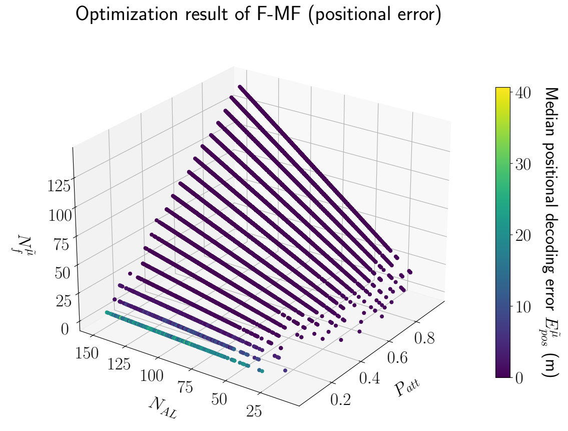

Figure S5a

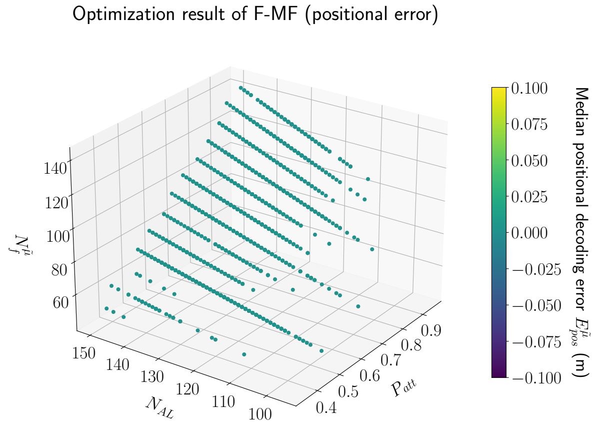

Figure S5b

Figure S5. Results of F-MF optimization after 3000 generations. Visualized on the axis are the overall number of attractors at all levels (N_{AL}), the probability of participation in an attractor (P_{att}) and the median number of fields ($N_f^{\tilde{\mu}}$). The colorbar shows the median positional decoding error ($E_{pos}^{\tilde{\mu}}$). The results are filtered by the median positional error: **a)** ($E_{pos}^{\tilde{\mu}} < \infty$), **b)** ($E_{pos}^{\tilde{\mu}} == 0.0$)

. There do exist some resulting models with $N_{AL} < 20$ in **a)**, although this is not clearly visible due to the 3D perspective.

We then calculated the Kullback-Leibler divergence (D) for the field size distribution (with a gamma distribution) and the field location distribution of each neuron per network. The mean (D^{μ}) and standard deviation (D^{σ}) of all networks is visualized in Figures S3a and S3b for the field sizes and locations, respectively.

The results shown in these figures do not indicate that there is any kind of pattern, convergence or correlation between the decoded positional error $E_{pos}^{\tilde{\mu}}$ of a network and its field location/size initialization. On the contrary, it seems like all networks, independently from their decoding accuracy, are similarly close to their respective true distribution. The small, overall divergence of the networks can be explained by the way the fields sizes and locations are initialized. The maximal accumulative field size $\bar{\Sigma}_{fs}$ restricts the number and sizes of the fields, especially when it is almost reached. We then try to find a size, based on the gamma distribution, which would still fit without exceeding $\bar{\Sigma}_{fs}$. We repeat this process for maximally N_{try} times, hence the actual field size distribution can diverge slightly from the original one. Since fields are not allowed to overlap, it can also happen, that the location has to be sampled many times before one without overlap is found. This can negatively impact the KL divergence from the uniform distribution.

The higher divergence of the field sizes of model B from the gamma distribution ($\alpha = 2.02, \theta = 1.8$) can be explained by the combination of a small $\bar{\Sigma}_{fs}$ with a gamma distribution that returns fields larger than $2.0\,\mathrm{m}$ in 70% of the cases. This can result in an overproportional number of small fields, relative to the original (gamma) distribution.

Overall we could not find any hint or reason, why a network with the same parameters can have such a high variance in its decoded positional accuracy. In order to investigate this further, one has to analyze the exact distribution of the field locations and their sizes, i.e. find patterns, groups, or other irregularities. We elaborate more on this topic further in the future work.

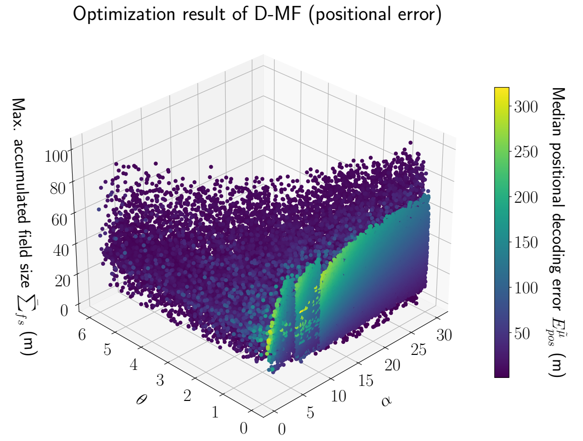

Figure S6a

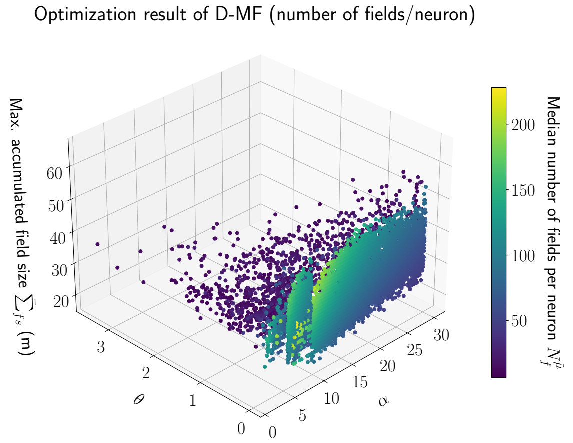

Figure S6b

Figure S6. Results of D-MF optimizations after 1600 generations. Visualized are the parameters of the network on the axis (α, θ and $\bar{\Sigma}_{fs}$) and on the colorbar the median positional error (**a**)) as well as the median number of fields per neuron (**b**)). The results are filtered by the median positional error: **a)** ($E^{\tilde{\mu}}_{pos} < 5.0$), **b)** ($E^{\tilde{\mu}}_{pos} < 1.0$)

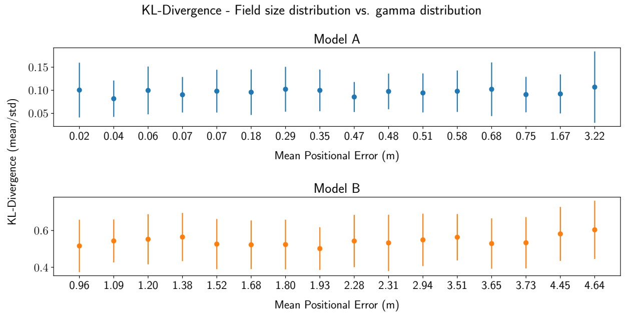

Figure S7a

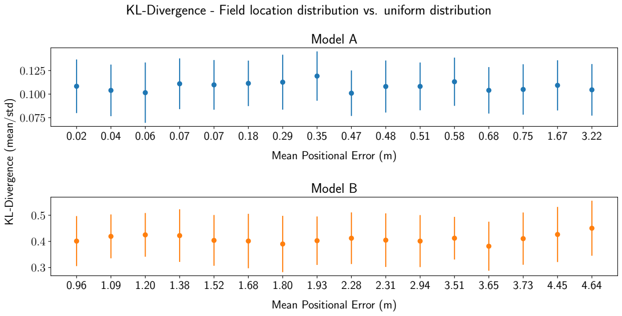

Figure S7b

Figure S7. The KL divergence of the field size distribution vs. the gamma distribution as well as the field location distribution vs. the uniform distribution used for sampling locations for

Table S5. Evaluated models.

ID	Network Type	α	θ	$\bar{\Sigma}_{fs}$	N_{run}	N^{μ}_{f}	$\bar{E}^{\mu}_{p}$	$\bar{E}^{min}_{p}$	$\bar{E}^{max}_{p}$
A	D-MF	0.66	1.8	59	16	37.1	0.41	0.02	3.22
B	D-MF	2.02	1.8	15	16	5.3	2.11	0.96	4.64

Table S6. Network and optimization parameters, as well as metrics.

Parameter	Description	Default value

**Global parameters**		
Network		
N_{neu}	Number of neurons	50
I_{bck}	Uniform background input	0.1
I_{loc}	Location specific input	0.05
W_{exc}	Maximum excitatory weight	0.7
W_{inh}	Minimum inhibitory weight	-0.15
P_{dro}	Percentage of drop-out (dead) neurons	–
Simulation		
T	Total simulation time (s)	20
τ	Membrane time coconstant	0.01
N_run	Number of simulation runs for one experiment	20
Environment		
L_{env}	Environment length (m)	200
L_{bin}	Lenght/size of a single bin (m)	0.5
N_{bins}	Number of bins in the environment	400
**F-MF specific parameters**		
P_{att}	Probability of attractor participation for one neuron	0.3
N_{AL_0}	Number of attractors in minimum layer	1
N_{AL_1}	Number of attractors in medium layer	2
N_{AL_2}	Number of attractors in maximum layer	5
L_{int}	Interaction length between neurons in one attractor	5%
$sample_{replace}$	Sample neurons for attractor w/o replacement	$False$
**D-MF specific parameters**		
α	Alpha of gamma-distribution	3.16
θ	Theta of gamma-distribution	1.8
TH_{fsr}	Threshold of the field size ratio	0.9 (90%)
P_{fc}	Field connection probability	0.9 (90%)
$\bar{\Sigma}_{fs}$	Maximum allowed sum of all field sizes of one neuron (m)	30
**Grid model specific parameters**		
N_{mod}	Number of modules	–
N_{neu}^{mod}	Number of neurons per module	–
S_{mod}	Scale of the modules	–

S_{mod}^{min}	First/minimal scale of all modules	–
**Metrics**		
$N_f^{\tilde{\mu}}$	Median number of fields per neuron	–
$E_{pos}^{\tilde{\mu}}$	Median error of multiple runs	–

REFERENCES
